# Clinical Factors and Income Status are Associated with Depression and Anxiety in Head and Neck Cancer Patients

**DOI:** 10.1177/19160216251398771

**Published:** 2026-03-19

**Authors:** Ashok R. Jethwa, Catriona M. Douglas, Katrina Hueniken, Geoffrey Liu, Andrew Bayley, Shao Hui Huang, Aaron Hansen, Douglas Chepeha, Leba M. Sarkis, David P. Goldstein, Madeline Li, Shayanne A. Lajud, John R. de Almeida

**Affiliations:** 1 Department of Otolaryngology-Head and Neck Surgery, University of Minnesota, Minneapolis, MN, USA; 2 Department of Otolaryngology-Head and Neck Surgery, Queen Elizabeth University Hospital, Glasgow, Scotland, UK; 3 Department of Biostatistics, Princess Margaret Cancer Centre, University of Toronto, Toronto, Ontario, Canada; 4 Department of Radiation Oncology, Sunnybrook Health Sciences Centre, Odette Cancer Centre, Toronto, ON, Canada; 5 Department of Radiation Oncology, Princess Margaret Cancer Centre, University of Toronto, Toronto, Ontario, Canada; 6 Department of Medical Oncology, Princess Margaret Cancer Centre, University of Toronto, Toronto, Ontario, Canada; 7 Department of Otolaryngology-Head and Neck Surgery, University of Michigan, Ann Arbor, Michigan, USA; 8 Department of Otolaryngology-Head and Neck Surgery, Princess Margaret Cancer Centre, University of Toronto, Toronto, ON, Canada; 9 Department of Psychosocial Oncology and Palliative Care, Princess Margaret Cancer Centre, University of Toronto, Toronto, Ontario, Canada; 10 Institute of Health Policy Management and Evaluation, University of Toronto, Toronto, Canada

**Keywords:** head and neck cancer, depression, anxiety, psychosocial distress, mental health

## Abstract

**Importance:**

Depression and anxiety affect a significant portion of head and neck cancer (HNC) patients. Furthermore, depression has been shown to result in decreased survival and worse functional outcomes.

**Objective:**

The purpose of this study is to determine the prevalence of depression and anxiety in a cohort of HNC patients at all subsites.

**Design:**

This is a retrospective review of prospectively gathered data in a cohort of patients aged 18 years of age and over, diagnosed with HNC, between August 2011 and December 2017, treated at the Princess Margaret Cancer Centre in Toronto, Canada.

**Setting/Participants:**

Adult Patients with a new diagnosis of HNC treated at the Princess Margaret Cancer Center/University Health Network between August 2011 and December 2017 were included in the study.

**Exposures:**

Patients were initially screened using the Direct Assessment and Response Tool (DART), which includes the Edmonton Symptom Assessment Scale (ESAS) to identify psychosocial distress.

**Main outcome measures:**

If positive on DART, patients were then asked to complete the Generalized Anxiety Disorder 7-Item Scale and the Patient Health Questionnaire-9. Associations with anxiety/depression and variables were assessed using univariate and multivariate logistic regression

**Results:**

A total of 586 patients were included in our study. The median age of the group was 60 (22-89 years), with a male predominance (78.2%). Most patients (78.8%) had advanced disease at diagnosis. The oropharynx was the most common site (46.5%). The prevalence of depression and anxiety was 13.1% and 9.9%, respectively. Multivariate analysis showed that nonmarried status, non-white ethnicity, disease progression, lower income, and increased out-of-pocket costs were associated with depression. Definitive surgery, ECOG ≥1, and lower income were associated with anxiety. Disease stage, smoking/drinking history, and HPV status were not associated with anxiety/depression.

**Conclusions:**

A portion of patients are at higher risk of developing sustained depression and anxiety at 12 months following treatment, which may be predicted early, facilitating early mental health intervention.

**Relevance:**

Identifying factors associated with depression and anxiety in HNC patients will aid in providing early intervention.

## Graphical abstract

**Graphical Abstract:**
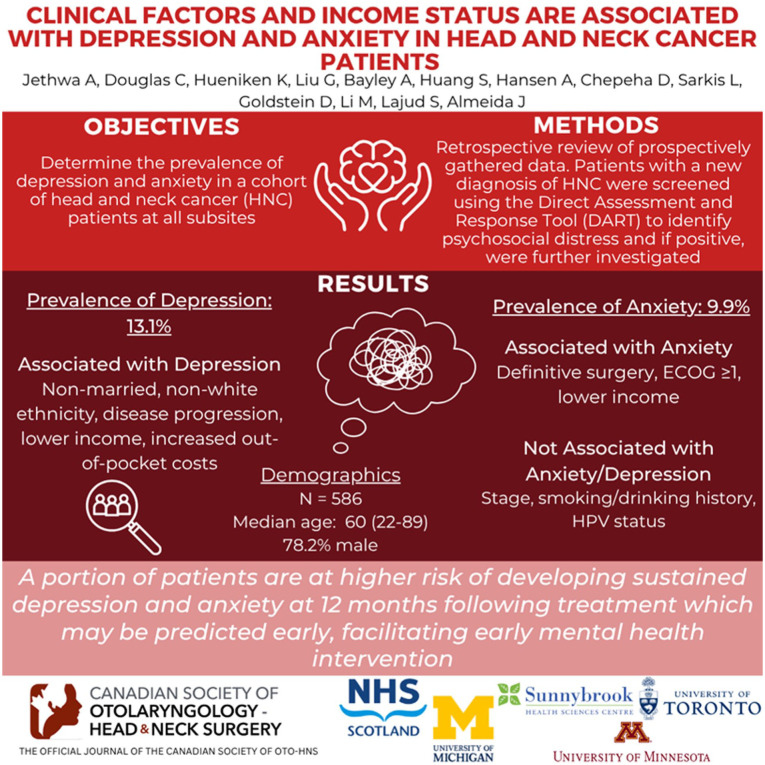

## Key Messages

- There is significant heterogeneity in studies assessing both the incidence and prevalence of depression in head and neck cancer patients.
- In our study of 586 patients, the prevalence of depression and anxiety was 13.1% and 9.9% respectively. Multivariable analysis showed that nonmarried status, non-white ethnicity, disease progression, lower income, and increased out-of-pocket costs were associated with depression. Definitive surgery, ECOG ≥1, and lower income were associated with anxiety.
- A portion of patients are at higher risk of developing sustained depression and anxiety at 12 months following treatment, which may be predicted early, facilitating early mental health intervention during treatment planning.

## Introduction

Head and Neck cancer is a severely debilitating disease that affects the inherent primitive functions of swallowing, phonation, mastication, taste, smell, and breathing, which significantly impairs overall quality of life, placing a cumbersome burden on both the patient and their carer. It is the seventh most common cancer worldwide and both its incidence and mortality are on the rise, particularly in young female patients with a predicted 30% annual increase in incidence in certain regions by 2030.^1^ A review of the literature demonstrates that depression may affect up to 48% of patients with head and neck cancer,^2^ with the oral cavity/pharynx and laryngeal primary sites demonstrating the third and fourth highest rates of suicide among all cancers, respectively. Unfortunately, however, there is significant heterogeneity in studies assessing both the incidence and prevalence of depression in head and neck cancer patients, limited by small sampling sizes, single institution series, and retrospective reporting without the use of objective, validated questionnaires. Thus, identifying patients at high risk of emotional distress preoperatively remains challenging.

Psychosocial care has become an integral component of cancer survivorship, and in 2004, the Canadian Federal Government’s public health agency introduced emotional distress as the sixth “Vital sign” with the routine screening for emotional distress now being endorsed by several international guidelines, including the National Comprehensive Cancer Network (NCCN).^3^ There is no current gold standard screening process that has been widely adopted; however, patient validated, objective screening systems have been developed in Toronto, Canada, namely the distress assessment and response tool (DART), which includes the Generalized Anxiety Disorder 7-Item Scale (GAD-7) and the Patient Health Questionnaire (PHQ-9) to specifically assess anxiety and depression, respectively. The Edmonton Symptom Assessment Scale (ESAS)^4^ is also a component of DART, providing a broader self-reported symptoms intensity tool. The GAD-7 is a 7-item scale whereby a cut-off score of ≥10 has a sensitivity of 89% and specificity of 82% for identifying anxiety compared to the gold standard of a mental health professional interview, highlighting its efficacy.^5^ Similarly, a cut-off score of ≥10 on the PHQ-9 has a sensitivity of 82% and specificity of 88% for diagnosing depression.^6^ Thus, the utility and efficacy of these secondary questionnaires have been well established, and specifically in head and neck cancer patients. The purpose of this study is to implement these validated objective patient and clinician-reported screening tools to determine the prevalence of both depression and anxiety in a large cohort of patients with head and neck cancer treated at a tertiary referral center. Further to this, we aimed to identify any patient or disease factors that predict anxiety or depression, facilitating early intervention in future treatment paradigms.

## Methods

### Sample

**Figure 1.**
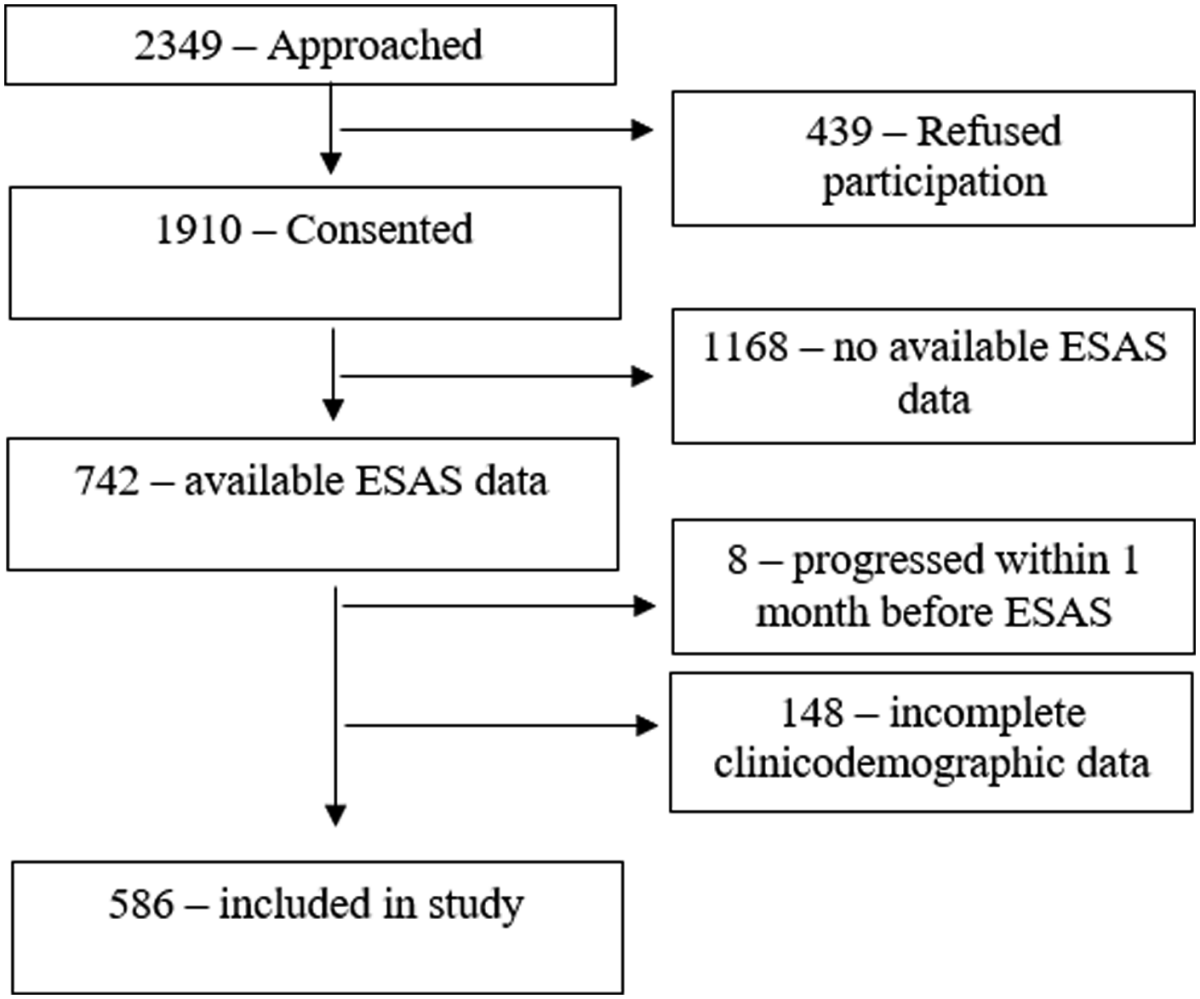
Flow Diagram depicting recruitment for 586 Adult patients with Head and Neck Cancer.

This is a retrospective review of prospectively gathered data in a cohort of patients aged 18 years of age and over, diagnosed with head and neck cancer, between August 2011 and December 2017, treated at the Princess Margaret Cancer Centre in Toronto, Canada. Research ethics board approval was obtained by the University Health Network ethics board committee. A summary of the recruitment process has been provided in Figure 1. In brief, patients were recruited who agreed and consented to participate in the study and completed the Edmonton Symptoms Assessment tool (ESAS).^5^ Baseline demographic data were collected prior to treatment, followed by patient-reported surveys to assess both depression and anxiety performed between 10 and 14 months following the completion of treatment, from October 2012 to December 2018. Patients with cancers of all histological subtypes of the oropharynx, larynx, lip or oral cavity, nasopharynx, and hypopharynx, as well as head and neck cancers with an unknown primary, were eligible for this study. The AJCC staging version 7 was used at the time of data collection.

### Measures

Self-reported patient information included race, marital status, employment status, living arrangements, and income at baseline; monthly out-of-pocket costs during treatment; and lost income, as well as speech and swallowing function at 12 months post-treatment. All other clinical variables used in this study were clinician-reported or collected following an assessment of medical records.

Following the conclusion of treatment, patients were initially screened for depression and anxiety (referred to together as “distress”) using the DART as previously described.^7^ The ESAS tool, an instrument that asks patients to rate the severity of various symptoms for that day, including anxiety and depression, was also completed as a component of the DART, and patients who scored 3 or greater for anxiety and 2 or greater for depression were considered at risk of affective disorders. Patients who screened positive for anxiety were then prompted to complete the GAD-7,^5^ and those who screened positive for depression completed the PHQ-9.^6^ Scores of ≥10 on each inventory were considered to be indicators of moderate-to-severe distress that requires intervention, while scores of 5-9 indicated mild distress. The same screening tools were applied to 306 patients at baseline, within 60 days of diagnosis.

### Statistical Analyses

The prevalence of anxiety and depression post-treatment was reported based on GAD-7/PHQ-9 thresholds of 5-9 and ≥10, respectively, for mild and moderate-to-severe distress with 95% confidence intervals.

For our main analysis, unadjusted associations between clinicodemographic variables and significant distress (measured as GAD-7 or PHQ-9 score ≥ 10 for anxiety and depression, respectively) were computed using univariable logistic regression. Clinicodemographic variables included in our main analysis were age at diagnosis, sex, ethnicity, marital status, living arrangements, employment status, stage, disease site, treatment modality, smoking, drinking, ECOG performance status, and any disease progression from diagnosis up to 1 month before the ESAS questionnaire was completed. Multivariable logistic regression was used to describe adjusted associations with distress. Backwards model selection was performed to reduce the number of variables in multivariable analysis, with a stay criterion of 0.05.

Secondary analyses were performed on a smaller subset of patients with available data for calculating baseline income, lost income due to cancer, and mid-treatment out-of-pocket costs due to cancer and functional status (speaking in public, eating in public, and swallowing). Income data was categorized as < 40,000 CAD, 40-79,000 CAD, or ≥80,000 CAD. Out-of-pocket costs were collected as part of a post-surgical questionnaire. Associations between these variables and distress were assessed by unadjusted logistic regression. The association between functional status and distress was explored with frequency tables and Fisher’s exact tests.

All tests were two-sided, with the α for significance set at .05. Data analyses were performed using SAS version 9.4.

## Results

### Patient Demographics

In total, 586 patients were included who had completed the DART following treatment and during the study period, and 306 patients completed the DART at baseline. The median age at diagnosis was 60 years (range 22-89 years) with a male-to-female ratio of 3.6:1. 462 (79%) patients had advanced stage disease (stage III or IV) at the time of diagnosis. The oropharynx was the most common subsite (46.5%), followed by the lip and oral cavity. (21.5%). Two-thirds of the patients were smokers. Almost half (45.1%) of patients were treated with chemoradiotherapy, and 136 patients (23.1%) underwent definitive surgery. A summary of patient characteristics, including treatment modality, has been provided in Table 1.

**Table 1.** Patient Characteristics of Study Population.

Variable		**Overall**
Age at diagnosis (years), median (range)		60 (22-89)
Sex, n (%)	Female	128 (21.8)
	Male	458 (78.2)
Stage, n (%)	I-II	124 (21.2)
	III-IV	462 (78.8)
Subsite, n (%)	Larynx	92 (15.6)
	Lip and oral cavity	126 (21.5)
	Oropharynx	273 (46.5)
	Other/unknown primary	95 (16.2)
Race, n (%)	Non-white	108 (18.4)
	White	478 (81.6)
Marital status, n (%)	Married/common law	423 (72.2)
	Not married	163 (27.8)
Currently working, n (%)	Yes	325 (55.5)
	No	261 (44.5)
Treatment modality, n (%)	Radiation/CRT	450 (76.7)
	Definitive surgery (± adjuvant treatment)	136 (23.2)
Drinking, n (%)	Ever alcohol use	387 (66.0)
	Never alcohol use	199 (34.0)
Smoking, n (%)	Ever smoker	388 (66.2)
	Never smoker	198 (33.8)
Performance status, n (%)	ECOG 0	376 (64.2)
	ECOG ≥1	210 (35.8)
Progressed before ESAS, n (%)	No	549 (93.7)
	Yes	37 (6.3)
Income (CAD), n (%)	Missing	447
	<40k	32 (23.0)
	40-79k	41 (29.5)
	80k+	66 (47.5)
Out-of-pocket costs ($), median (range)		420 (0-7120)

Abbreviations: ECOG, Eastern Cooperative Oncology Group; CAD, Canadian Dollars.

### Prevalence of Depression and Anxiety

At baseline in 306 patients, 116 (38%) had an ESAS anxiety score ≥ 3 with a median GAD score of 7 (0-21). A total of 116 (38%) patients had an ESAS depression score ≥ 2 with a median PHQ score of 5 (0-21). Of the 586 patients who completed the DART, 202 (34.5%) screened positive for depression, and 148 (25.3%) of patients screened positive for anxiety. A total of 75/202 (37.1%) patients who screened positive on the DART for depression did not go on to complete the PHQ questionnaire. Similarly, 65/148 (43.9%) of patients who screened positive on DART did not go on to complete the GAD-7. There were a total of 40/586 patients (6.8%) of patients who completed the DART and had a PHQ score of 5 to 9, indicating mild depression, and 37/586 patients (6.3%) with a PHQ score of greater than or equal to 10, indicating moderate-to-severe depression. There was a total of 37/586 (6.3%) patients who screened positive on DART and had a GAD-7 anxiety score of 5 to 9, indicating mild levels of anxiety. A total of 21/586 (3.6%) patients achieved a score of greater than or equal to 10 on the GAD-7, indicating severe anxiety. This resulted in a total of 77/586 (13.1%) of patients experiencing mild-to-severe depression and 58/586 (9.9%) of patients experiencing at least mild-to-severe levels of anxiety. A total of 47/586 (8%) of patients experienced both depression and anxiety following treatment. These results have been summarized in Tables 2 and 3.

**Table 2.** Baseline and Post-Treatment Anxiety and Depression Scores in 306 and 586 Patients, Respectively.

	Post-Treatment (n = 586)	Baseline (n = 306)	*P*-value
ESAS Anxiety ≥3, n (%)	105 (18)	116 (38)	<.001
GAD score*, median (range)	6.5 (0, 21)	7 (0, 21)	
GAD 5+, n (%)	58 (9)	65 (21)	<.001
GAD 10+, n (%)	21 (4)	23 (8)	.017
ESAS Depression ≥2, n (%)	93 (16)	115 (38)	.019
PHQ score*, median (range)	7 (0, 21)	5 (0, 21)	
PHQ 5+, n (%)	77 (13)	49 (16)	.24
PHQ 10+, n (%)	37 (6)	20 (7)	.95

* Median GAD and PHQ scores are calculated only from patients who screened positive for anxiety or depression (respectively) on the ESAS.

**Table 3.** A Summary of the Results of 586 Patients Who Completed the DART Screening Questionnaire.

GAD Score (Categories)	PHQ Score (Categories)					
	Screened negative for Depression (ESAS 0-1)	Screened positive for Depression, missing PHQ	PHQ 0-4	PHQ 5-9 (Mild Depression)	PHQ ≥ 10 (Moderate-Severe Depression)	Total
Screened negative for Anxiety (ESAS 0-2)	384 (66)	24 (4)	13 (2)	13 (2)	4(7)	438 (75)
Screened Positive for Anxiety, Missing GAD	12 (2)	51 (9)	1 (<1)	0-	1(<1)	65
GAD 0-4	6 (1)	0	7(1)	10 (2)	2 (<1)	25 (4)
GAD 5-9 (Mild Anxiety)	6 (1)	0	1(<1)	15 (3)	15 (3)	37 (6)
GAD ≥ 10 (Moderate-Severe Anxiety)	2 (<1)	0	2(<1)	2(<1)	15 (3)	21 (4)
Total	410 (70)	75 (13)	24 (4)	40 (7)	37 (6)	586

A GAD-7 score was performed on patients who scored ≥ 3 and a PHQ was obtained on those who scored ≥2 to measure anxiety and depression, respectively.

Numbers with percentage of total in parenthesis.

### Univariable Analysis

Univariate analysis demonstrated that non-white ethnicity (OR = 2.6, p = 0.009) and disease progression (OR = 4.0, *P* = .002) were associated with a higher rate of depression. Patients with an ECOG score ≥1 had a significantly higher rate of anxiety (OR = 3.0 *P* = .015) and HPV status was not associated with anxiety (OR = 0.6, *P* = .311) or depression (OR = 0.5, *P* = .072). The univariate analysis has been provided in Table 4.

**Table 4.** Univariate Analysis of GAD ≥ 10 and PHQ ≥ 10.

n = 586		Anxiety (GAD ≥ 10), n = 21			Depression (PHQ ≥ 10), n = 37		
Variable	Level	N (%) with Anxiety	OR (95% CI)	*P*	N (%) with Depression	OR (95% CI)	*P*
Age at diagnosis			0.978 (0.938-1.018)	.286		0.978 (0.948-1.009)	.168
Sex	Male	17 (3.7)	Ref		27 (5.9)	Ref	
	Female	4 (3.1)	0.836 (0.276-2.532)	.753	10 (7.8)	1.352 (0.636-2.874)	.432
Stage	I-II	2 (1.6)	Ref		8 (6.5)	Ref	
	III-IV	19 (4.1)	2.616 (0.601-11.38)	.200	29 (6.3)	0.971 (0.432-2.181)	.943
Disease Site	Oropharynx	8 (2.9)	Ref		14 (5.1)	Ref	
	Larynx	3 (3.3)	1.116 (0.289-4.300)	.873	6 (6.5)	1.290 (0.481-3.463)	.612
	Lip and oral cavity	6 (4.8)	1.656 (0.562-4.878)	.360	9 (7.1)	1.423 (0.598-3.381)	.424
	Other/unknown primary	4 (4.2)	1.456 (0.428-4.949)	.547	8 (8.4)	1.701 (0.690-4.192)	.248
Ethnicity	White	16 (3.3)	Ref		24 (5)	Ref	
	Non-white	5 (4.6)	1.401 (0.502-3.913)	.519	13 (12)	2.588 (1.272-5.266)	.009
Marital Status	Married/common law	12 (2.8)	Ref		22 (5.2)	Ref	
	Not married	9 (5.5)	2.001 (0.827-4.844)	.124	15 (9.2)	1.847 (0.933-3.656)	.078
Currently Working	No	12 (3.7)	Ref		24 (7.4)	Ref	
	Yes	9 (3.4)	0.931 (0.386-2.245)	.875	13 (5)	0.657 (0.327-1.318)	.237
Treatment Modality	Chemo/CRT	12 (2.7)	Ref		26 (5.8)	Ref	
	Surgery ±adjuvant	9 (6.6)	2.586 (1.065-6.277)	.036	11 (8.1)	1.435 (0.689-2.985)	.334
Drinking	Never alcohol user	7 (3.5)	Ref		10 (5)	Ref	
	Ever alcohol user	14 (3.6)	1.029 (0.408-2.593)	.951	27 (7)	1.417 (0.671-2.990)	.360
Smoking	Never smoker	6 (3)	Ref		12 (6.1)	Ref	
	Ever smoker	15 (3.9)	1.286 (0.491-3.369)	.608	25 (6.4)	1.067 (0.524-2.172)	.857
ECOG Performance Status	ECOG 0	8 (2.1)	Ref		19 (5.1)	Ref	
	ECOG 1+	13 (6.2)	3.035 (1.237-7.448)	.015	18 (8.6)	1.761 (0.903-3.435)	.097
Disease progression	No	19 (3.5)	Ref		30 (5.5)	Ref	
	Yes	2 (5.4)	1.593 (0.356-7.119)	.542	7 (18.9)	4.036 (1.639-9.941)	.002

### Multivariable Analysis

Nonmarried status (OR = 2.1 *P* = .045) and non-white ethnicity (OR = 2.5, *P* = .013) were significantly associated with depression. Disease progression was significantly associated with depression (OR = 3.6, *P* = .007), but not anxiety. There was a significant association with anxiety and the treatment modality patients received; definitive surgery was significantly associated with higher odds of anxiety (OR = 2.9, *P* = .022). ECOG performance status greater than zero was also significantly associated with anxiety (OR = 3.3, *P* = .010). The results of the multivariate analysis are provided in Table 5.

**Table 5.** Multivariate Analysis of GAD ≥ 10 and PHQ ≥ 10.

Odds Ratio Estimates, Anxiety GAD ≥ 10			Odds Ratio Estimates, Depression PHQ ≥ 10		
Effect	Point Estimate (95% Wald Confidence Limits)	*P*-value	Effect	Point Estimate (95% Wald Confidence Limits)	*P*-value
Treatment Modality Surgery ± adjuvant vs Chemo/CRT/RT	2.854 (1.162-7.011)	.0221	Ethnicity Non-white vs White	2.548 (1.223-5.308)	.0125
ECOG Performance Status ECOG 1+ vs ECOG 0	3.278 (1.324-8.113)	.0103	Marital Status Not Married vs Married/Common Law	2.051 (1.017-4.137)	.0448
Age, sex, ethnicity, marital status, employment, smoking, drinking, stage at diagnosis, disease site, and previous disease progression removed during backwards model selection.			Previous Disease Progression Yes vs No	3.584 (1.418-9.058)	.007
			Age, sex, employment, smoking, drinking, stage at diagnosis, ECOG performance status, disease site, and treatment modality removed during backwards model selection.		

### Income and Out-of-Pocket Costs

Income data were available for 206 patients. The majority of patients (104, 50.5%) reported an income of ≥80,000 CAD, with 57 (27.7%) patients reporting an income of 40-79,000 CAD and 45 (21.8%) reporting an income of < 40, 000 CAD (Table 6). Patients with lower income (<40,000 CAD) were more likely to develop depression (OR = 1.161, *P* = .0024) and anxiety (OR = 1.132, *P* = .0016) (Table 7). Increased out-of-pocket costs were also associated with depression (OR = 1.068, *P* = .0219).

**Table 6.** Income Data in Patients with GAD-7 and PHQ-9 ≥10.

ANXIETY, GAD ≥10	Income Category				DEPRESSION PHQ ≥10	Income Category			
	80k+	40-79k	<40k	Total		80k+	40-79k	<40k	Total
No	101	50	37	188	No	103	53	39	195
	53.72	26.6	19.68			52.82	27.18	20	
	97.12	87.72	82.22			99.04	92.98	86.67	
Yes	3	7	8	18	Yes	1	4	6	11
	16.67	38.89	44.44			9.09	36.36	54.55	
		2.88	12.28	17.78			0.96	7.02	13.33
Total	104	57	45	206	Total	104	57	45	206

**Table 7.** Univariate Analysis of Income Data, OOPC, and Lost Income.

Parameter	Level	Reference	Anxiety (GAD ≥ 10)				Depression (PHQ ≥ 10)			
			Odds Ratio	Lower 95% CI	Upper 95% CI	*P*-value	Odds Ratio	Lower 95% CI	Upper 95% CI	*P*-value
Income	40-79k	80k+	1.062	0.990	1.140	.0938	1.099	1.005	1.201	.0385
Income	<40k		1.132	1.048	1.222	.0016	1.161	1.054	1.278	.0024
Mid-Treatment OOPC* (Thousands)			0.963	0.915	1.013	.1413	1.068	1.010	1.130	.0219
Lost Income (Thousands)			0.933	0.969	1.018	.583	0.997	0.980	1.015	.7646

Abbreviations: OOPC, out-of-pocket costs.

### Functional Status

There was no significant association between depression/anxiety and speaking in public, the ability to eat in public, or nutritional mode.

## Discussion

Given that patients being treated for head and neck cancer are living longer, there exists a growing need to identify those at most at risk of psychological distress to preemptively commence appropriate mental health support and intervention. The results of our cohort study indicate that at approximately 12 months following treatment, at least 13.1% of patients experience depression, 10% of patients experience anxiety and 8% of patients will experience both. A recent cohort study of 71 patients undergoing treatment for head and neck cancer reported an overall prevalence of depression of 51% and anxiety of 46% also using the GAD-7 score and PHQ-9 self-administered questionnaire, albeit all patients in that study had undergone both surgery and free flap reconstruction.^8^ In comparison, van Beek et al^9^ recently provided the results of a longitudinal study of 345 patients who underwent chemoradiation for head and neck cancer, resulting in anxiety in 11% of patients at 24 months and depression in 7% of patients at 12 months. This is in keeping with the results of our study, considering more than three-quarters of our patient cohort underwent chemoradiation. The higher rates of depression and anxiety seen in the aforementioned surgically treated cohort who had undergone free flap surgery may at least in part be explained by a higher portion of patients having advanced disease, with two-thirds of their patients having stage IV disease, along with the visible facial disfigurement and dysfunction that results from ablative surgery. Further, to this, these patients are also likely to have received adjuvant chemoradiation given the advanced stage of their disease, magnifying the side effects of multimodality therapy, although this is not clearly indicated in their data. Thus, the results of these studies highlight the potentially significant disease burden of mental health on this disease population.

Previous studies have attempted to define the population group at highest risk of developing sustained depression following treatment. Haisefield-Wolfe et al^10^ in their systematic review of patients with head and neck cancer established that being male, unmarried, having less education, a history of smoking, age less than 40, and having lower physical functioning status are associated with depression. In contrast to this, and more recently, Korsten et al^11^ in their systematic review of the literature found that there was strong evidence that preoperative depression was significantly associated with depression in head and neck cancer patients, and that all other factors including age, marital status, education, ethnicity, smoking and alcohol status and tumor location did not consistently correlate well with depression. Such contrasting results have at least in part occurred due to the heterogeneity and lack of objectively validated reporting among previously published series, as was highlighted by the authors. The results of our study indicate that non-white patients, single patients, those who had disease progression, lower annual income status, and those with increased out-of-pocket costs were more likely to have moderate-to-severe depression at approximately 12 months following treatment. It is not surprising that unmarried patients were more likely to have depression, which has been previously well established at other cancer disease subsites and the general psychiatric population,^12^ likely related to the lack of social support in managing the detrimental dysfunction incurred with treatment, further exacerbated by the fact that male patients are often less likely to seek social support. Patients with lower annual income and those with higher out-of-pocket costs were also more likely to experience depression, which at least in part relates to their inability to return to work in their premorbid state,^13^ compounding the financial stress during both the active and recovery phases of treatment. Interestingly, untreated depression has also been previously demonstrated to affect wound healing, appetite, and compliance with treatment.^14^ Therefore, identifying potential risk factors for depression is imperative for clinicians treating head and neck cancer.

Similarly, single institutional studies^7^ have suggested that gender, age, tumor stage, and health-related quality of life are associated with anxiety in head and neck cancer patients, with younger female patients experiencing more anxiety than men.^15,16^ Our findings are significant in that they indicate that both definitive surgery and ECOG performance status both attributed to higher levels of anxiety on multivariate analysis. Patients with higher ECOG performance status often have multiple other significant comorbidities, further exacerbating the overall chronic disease burden. Chronic medical illness is in itself associated with anxiety that can be related to biologic effects of the illness or the behavioral limitations imposed by the disease, such as decreased ability to complete activities of daily living, which can heighten anxiety.^17^ Anxiety in head and neck cancer is less well understood and investigated than depression. However, it is reported that between 6-34% of patients with cancer experience anxiety.^18^ Similar to depression, primary surgical treatment imparts cosmetic deformity and functional impairment, resulting in passivity and social isolation, particularly in young female patients, which can often be understated. Further to this, recent studies have highlighted the temporal course of anxiety symptoms during treatment, highest at the pretreatment phase in up to 29% of patients, which declines to 10% at 24 months.^7^ Thus, the results of our study in combination with previous findings support the notion of offering psychosocial support pre-emptively in younger patients undergoing primary surgical treatment at the preoperative phase.

The strengths of this study lie in the fact that it represents a large cohort of patients of head and neck cancer across all subsites who have undergone both surgery and chemoradiation with an assessment of both anxiety and depression, therefore providing a true representation of patients encountered in a real-world clinical setting. Furthermore, we have utilized the DART screening tool, a comprehensive screening system that utilizes both the PHQ-9 for depression and the GAD-7 for anxiety, enabling a unique opportunity to concurrently administer distress tools that have previously been well validated and specific to head and neck cancer.^19^ Thus, our results have identified clinically relevant risk factors that can be acted upon by the treating team preoperatively to predict patients at higher risk of emotional distress during treatment. One major limitation of this study, although not unique to this psychosocial study, is the use of a surrogate patient-reported outcome as the standard for distress diagnosis. While the sensitivity and specificity are quite high for these questionnaires, these instruments are not the standard for establishing psychiatric diagnoses. Various screening tools have been used in previous studies to identify depression and anxiety, and currently, there is no established consensus on which screening tool evaluates the most ideal psychometric symptoms. Further to this, ideally, we would have performed a longitudinal study comparing the preoperative distress screening scores along a continuum of different phases of treatment, as has been previously demonstrated. However, the primary outcome in this study was to identify the risk factors for both depression and anxiety at 12 months following treatment, which provides time for the acute toxicities relating to treatment to subside. Despite the prospective nature of the study, the completion rate for the GAD-7 and PHQ-9 by patients was poor. Approximately 40% of patients did not complete the GAD-7 and PHQ-9 after screening positive for distress on the ESAS. It is conceivable that those patients who did not complete these instruments may have systematically biased the results, particularly if they were preferentially depressed or anxious; however, we would argue that this would suggest an underestimate of the true prevalence of depression and anxiety in this cohort given the patients who were positive on DART, yet did not complete the GAD-7 and PHQ-9, yet were still included in the denominator. Similarly, we had a small sample size to evaluate speech/swallowing outcomes, as there were limited patients with poor functional outcomes. Therefore, it is certainly feasible that our results provide an underestimate of the true prevalence of depression and anxiety in head and neck cancer patients.

The results of this study indicate that patients who are non-white, unmarried, who had disease progression, lower annual income, or increased out-of-pocket costs had higher rates of moderate-to-severe depression. Patients with poorer ECOG performance status, who had definitive surgery, or had a lower annual income, had higher rates of moderate-to-severe anxiety. This further adds to the growing body of literature in identifying patients at high risk of depression and anxiety during treatment and arms the clinician with invaluable information that can direct early psychosocial intervention in specific patient groups.

## Conclusion

Patients with HNC are at risk of developing depression/anxiety. Several predictors of distress include non-white patients, unmarried patients, those who had disease progression, lower annual income, increased out-of-pocket costs, poorer ECOG performance status, and definitive surgery. High-risk patient populations for depression and anxiety may be candidates for early mental health evaluation.
